# The role of interleukin-1 in the pathogenesis of human Intervertebral disc degeneration

**DOI:** 10.1186/ar1732

**Published:** 2005-04-01

**Authors:** Christine Lyn Le Maitre, Anthony J Freemont, Judith Alison Hoyland

**Affiliations:** 1 Division of Laboratory and Regenerative Medicine, School of Medicine, University of Manchester, Manchester, UK

## Abstract

In this study, we investigated the hypotheses that in human intervertebral disc (IVD) degeneration there is local production of the cytokine IL-1, and that this locally produced cytokine can induce the cellular and matrix changes of IVD degeneration. Immunohistochemistry was used to localize five members of the IL-1 family (IL-1α, IL-1β, IL-1Ra (IL-1 receptor antagonist), IL-1RI (IL-1 receptor, type I), and ICE (IL-1β-converting enzyme)) in non-degenerate and degenerate human IVDs. In addition, cells derived from non-degenerate and degenerate human IVDs were challenged with IL-1 agonists and the response was investigated using real-time PCR for a number of matrix-degrading enzymes, matrix proteins, and members of the IL-1 family.

This study has shown that native disc cells from non-degenerate and degenerate discs produced the IL-1 agonists, antagonist, the active receptor, and IL-1β-converting enzyme. In addition, immunopositivity for these proteins, with the exception of IL-1Ra, increased with severity of degeneration. We have also shown that IL-1 treatment of human IVD cells resulted in increased gene expression for the matrix-degrading enzymes (MMP 3 (matrix metalloproteinase 3), MMP 13 (matrix metalloproteinase 13), and ADAMTS-4 (a disintegrin and metalloproteinase with thrombospondin motifs)) and a decrease in the gene expression for matrix genes (aggrecan, collagen II, collagen I, and SOX6).

In conclusion we have shown that IL-1 is produced in the degenerate IVD. It is synthesized by native disc cells, and treatment of human disc cells with IL-1 induces an imbalance between catabolic and anabolic events, responses that represent the changes seen during disc degeneration. Therefore, inhibiting IL-1 could be an important therapeutic target for preventing and reversing disc degeneration.

## Introduction

Low back pain is a common, debilitating, and economically important disorder. Current evidence implicates loss of intervertebral disc (IVD) matrix consequent upon disc 'degeneration' as a major cause of low back pain [1]. Although many treatments aimed at relieving back pain are directed towards the degenerate IVDs (e.g. removal of protruding disc material, disc replacement, etc.), none of these are aimed at the processes of degeneration. Modern advances in therapeutics, particularly cell and tissue engineering, offer potential methods for inhibiting or reversing IVD degeneration that have not previously been possible, but they require a level of understanding of the pathobiology of degeneration of the IVDs that is not currently available [2].

Degeneration is characterized by increased degradation of the normal IVD matrix by locally produced matrix metalloproteinases (MMPs) and ADAMTS (a disintegrin and metalloproteinase with thrombospondin motifs) [3-6]. In addition, the nature of the matrix produced in the degenerate IVDs differs from that in normal IVDs, as a consequence of switches in the production of collagen within the inner annulus fibrosus (IAF), and nucleus pulposus (NP) from type II to type I [7] and in the synthesis of proteoglycan from aggrecan [8] to versican, biglycan, and decorin [9,10]. The resultant changes within the extracellular matrix have a number of consequences, resulting in loss of structural integrity, decreased hydration, and a reduced ability to withstand load.

Similar matrix changes have been reported in articular cartilage in osteoarthritis [11,12]. In this disease, the body of evidence points towards these being part of a more profound change in chondrocyte biosynthesis [13] driven by local production of IL-1 and tumour necrosis factor α [14-17]. Despite the similarities between IVD degeneration and the cartilage changes in osteoarthritis, there has been relatively little interest in exploring the possibility that the disease processes involved in IVD degeneration might be driven by similar alterations in local tissue cytokine biology, and particularly by IL-1 and tumour necrosis factor α. TNF α has been implicated in disc herniation and sciatic pain [18-21], but not in disc degeneration. There is, however, some circumstantial evidence implicating IL-1 in human IVD degeneration [22-26]. This evidence comes from studies on annulus fibrosus (AF) cells from rabbit IVDs [24,26,27] and NP cells from ovine [25] and rabbit IVDs [28], which suggest that IL-1 may have similar effects on the chondrocyte-like cells of IVDs to those seen in articular chondrocytes. IL-1 has been identified in herniated, displaced human discal tissue [23,29,30] but has not been investigated within the degenerate IVDs themselves. Two recent genetic studies suggest that IL-1 gene cluster polymorphisms contribute to the pathogenesis of lumbar IVD degeneration and low back pain [31,32]. Despite these data, there is no clear evidence that IL-1 is synthesized by native human disc cells (as opposed to cells within herniated disc tissue) or whether it can induce the altered synthesis of matrix molecules and degrading enzyme production by human IVD cells characteristic of IVD degeneration, particularly in the NP, where degenerative changes first appear.

This study investigates two hypotheses: that in human IVD degeneration, there is local production of the cytokine IL-1 by native disc cells, and that locally produced IL-1 can induce the cellular and matrix changes of IVD degeneration.

## Materials and methods

### Tissue samples

Human IVD tissue was obtained either at surgery or at post-mortem examination, with the informed consent of the patient or relatives. Local research ethics committee approval was given for this work by the following local research ethics committees: Salford and Trafford (Project number 01049), Bury and Rochdale (BRLREC 175(a) and (b)), Central Manchester (Ref No: C/01/008), and her Majesty's coroner (LMG/RJ/M6).

### Tissue samples for Immunohistochemical analysis

#### Post-mortem tissue

Preliminary studies from our laboratory (data not shown) have shown that IVD cells remain viable for at least 48 hours after death. We also have evidence that NP cells from retrieved cadaveric IVDs are biosynthetically identical to age-matched cells from non-cadaveric tissue, an observation borne out by others [4,33,34]. In all, eight discs recovered from six patients within 18 hours of death were used in this study (Table 1). They consisted of full-thickness wedges of IVD of 120° of arc removed anteriorly. This allowed well-orientated blocks of tissue incorporating AF and NP to be cut for histological study. The family practitioner's notes were examined for evidence of a history of sciatica sufficient to warrant seeking medical opinion, and such patients were excluded from the study.

#### Surgical tissue

Patients were selected on the basis of IVD degeneration diagnosed by magnetic resonance imaging and progression to anterior resection either for spinal fusion or disc replacement surgery to relieve chronic low back pain. Some patients underwent fusion at more than one level, because of instability. Occasionally the specimens retrieved from multilevel fusion included discs with low (0–3) degeneration scores (i.e. morphologically normal) at one level (Table 1) (The scoring system is described below). Wedges of disc tissue were removed in a manner similar to that described for cadaveric tissue.

#### Treatment of tissue specimens

A block of tissue incorporating AF and NP in continuity was fixed and processed into paraffin wax. As some specimens contained bone, all the samples were decalcified in ethylenediaminetetraacetic acid (EDTA) (we have previously shown that EDTA decalcification does not affect detectable levels of product using *in situ *hybridization or immunohistochemical staining [35] when compared to snap-frozen tissue). Sections from the tissue blocks were taken for H&E staining to score the degree of morphological degeneration according to previously published criteria [8]. This scoring system provided a representation of the grade of degeneration within a disc: scores of 0 to 3 represent a histologically normal (non-degenerate) disc; 4 to 6, histological evidence of low-level degeneration; 7 to 9, an intermediate degree of degeneration; and 10 to 12, severe degeneration. From this scoring, 30 discs were selected to represent a range of scores from non-degenerate (1 to 3) up to the most severe level of degeneration (12).

### Tissue samples for *in vitro *cell studies

Samples of degenerate IVD tissue (graded 6 to 10) were obtained from patients undergoing surgery for disc replacement for the treatment of chronic low back pain. Non-degenerate IVD tissue (graded 0 to 2) was also obtained from surgery for disc removal after trauma. Ten discs were used in triplicate for all treatments; all discs were lumbar in origin and the ages of the patients ranged from 18 to 44 years (mean 29.9).

### Production and localization of IL-1 family proteins

Immunohistochemistry was used to localize the two IL-1 agonists (IL-1α and IL-1β) and their antagonist IL-1Ra together with the active receptor IL-1RI (IL-1 receptor, type I) and the IL-1β-converting enzyme (ICE; caspase-1) within the 30 disc samples described in Table 1. In addition, rheumatoid synovium was selected as a positive control tissue for members of the IL-1 family. The immunohistochemistry protocol followed was as previously published [6]. Briefly, 4-μm wax sections were dewaxed and rehydrated, and endogenous peroxidase was blocked using hydrogen peroxide. Sections were washed in dH_2_O and then treated with chymotypsin enzyme antigen retrieval system (0.01% w/v chymotrypsin (Sigma, Poole, Dorset, UK), 20 min at 37°C) for IL-1α, IL-1β, IL-1Ra, and ICE. No enzyme retrieval was necessary for IL-1RI. After washing, non-specific binding sites were blocked at room temperature for 45 min, either with 20% w/v rabbit serum (Sigma), for IL-1Ra and IL-1RI, or with 20% w/v donkey serum (Sigma), for IL-1α, IL-1β, and ICE. Sections were incubated overnight at 4°C with mouse monoclonal primary antibodies against human IL-1Ra (1:200 dilution, R&D Systems, Abingdon, UK), IL-1RI (1:50 dilution, R&D Systems), and goat polyclonal primary antibodies against human IL-1α (1:300 dilution, Santa Cruz Biotechnology, Santa Cruz, CA, USA), IL-1β (1:300 dilution, SantaCruz), and ICE (1:10 dilution, SantaCruz). Negative controls in which mouse or goat IgGs (Dako, Cambridgeshire, UK) replaced the primary antibody (at an equal protein concentration) were used.

Following washes, sections reacted with mouse monoclonal antibodies were incubated in a 1:400 dilution of biotinylated rabbit anti-mouse antiserum (Dako), and sections reacted with goat polyclonal primary antibodies were incubated in a 1:300 dilution of biotinylated donkey anti-goat antiserum (Santa Cruz Biotechnology), all for 30 min at room temperature. Disclosure of secondary antibody binding was by the streptavidin-biotin complex (Dako) technique with 3,3'-diaminobenzidine tetrahydrochloride solution (Sigma). Sections were counterstained with Mayer's haematoxylin (Raymond A Lamb, East Sussex, UK), dehydrated, and mounted in XAM (BDH, Liverpool, UK).

#### Image analysis

All slides were visualized using a Leica (Leica, Cambridge, UK) RMDB research microscope and images captured using a digital camera and Bioquant Nova image analysis system (Bioquant, Nashville, TN, USA). Each section was divided into three areas of disc for the purposes of analysis – the NP, the Inner annulus fibrosus (IAF), and, where present, the outer annulus fibrosus (OAF) – and analysed separately. Within each area, 200 cells were counted and the number of immunopositive cells (brown-stained cells) expressed as a proportion of this. Averages and standard deviations were calculated for disc sections grouped with the scores 0 to 3, 4 to 6, 7 to 9, and 10 to 12. Data was then presented on graphs as means ± 2 standard errors to represent the 95% confidence intervals [36].

#### Statistical analysis

Data was non-parametric, and hence the Mann-Whitney U tests were used to compare the numbers of immunopositive cells in degenerate discs (groups 4 to 6, 7 to 9, and 10 to 12) with those in non-degenerate discs (scores 0 to 3). These tests were performed for each area of the disc analysed (i.e. NP, IAF, and OAF). In addition, the Wilcoxon paired samples tests were used to compare proportions of immunopositive cells in the different areas of the discs (i.e. NP vs IAF, NP vs OAF, and IAF vs OAF). This analysis was performed using all disc sections, regardless of level of degeneration.

### Investigation of the effect of IL-1 on human IVD cells in alginate culture

#### Issolation of Disc cells

Tissue samples were separated into NP and IAF tissue and transported to the laboratory in DMEM and Ham's F12 nutrient medium (DMEM + F12) (Gibco BRL, Paisley, UK) on ice. Tissue samples were finely minced and digested with 2 U/ml protease (Sigma) in DMEM + F12 media for 30 min at 37°C and washed twice in DMEM + F12. NP cells were isolated in 0.4 mg/ml collagenase type 1 (Gibco), and AF cells in 2 mg/ml collagenase type 1 (Gibco) for 4 hours at 37°C.

#### Alginate bead culture

It is well recognized that cells derived from the IVDs change their morphology and phenotype in monolayer culture, becoming similar to fibroblasts [37]. However, culturing the cells in systems such as alginate can restore the IVD cell phenotype [37]. We have therefore used cells in alginate gels to investigate the effects of IL-1 on cell behaviour. To achieve this, following isolation, cells were expanded in monolayer culture for 2 weeks, prior to trypsinization and resuspension in 1.2% medium-viscosity sodium alginate (Sigma) in 0.15 M NaCl at a density of 1 × 10^6 ^cells/ml and formation of alginate beads using 200 mM CaCl_2_. Following washes in 0.15 M NaCl, 2 ml of complete culture medium was then added to each well and cultures were maintained at 37°C in a humidified atmosphere containing 5% CO_2_. The culture medium was changed every other day.

#### Assessment of re-differentiated state in alginate

To ensure that the phenotype of cells treated with IL-1 were similar to the phenotype of cells within the IVDs in vivo, the cell phenotype was assessed in monolayer culture and at increasing times in alginate culture. The phenotype was then compared with that of uncultured, directly extracted cells. Phenotype was assessed using immunohistochemistry on cellular cytospins for directly extracted and monolayer cells, and wax-embedded alginate beads sectioned at 4 μm and mounted onto slides for analysis. Immunohistochemistry was performed for aggrecan, collagen type II, and collagen type I as described previously [38]. In addition, RNA was extracted from cells and reverse transcription performed using Avian Myeloblastosis Virus (AMV) reverse transcriptase (Roche, East Sussex, UK), and gene expression for the chondrogenic transcription factor SOX9 and the matrix constituents aggrecan, collagen II, and collagen type I were assessed (see below).

#### Image analysis

All slides were visualized using the Leica RMDB research microscope and images were captured using a digital camera and the Bioquant Nova image analysis system. Within each area, 200 cells were counted and the number of immunopositive cells was expressed as a proportion of this.

#### Statistical analysis

One-way ANOVA and Tukey post hoc tests were used to compare cellular gene expression of cells cultured in monolayer and alginate to uncultured, directly extracted cells. To perform this analysis, 2^-ΔCt ^(where Ct represents the cycle at which the set threshold is reached) for each sample was calculated to generate relative gene expression for each sample, including all control values. These values were then used in ANOVA and post hoc tests.

#### Treatment of cells with IL-1, RNA extraction, and cDNA formation

After 4 weeks in this culture system (the time required to allow redifferentiation to the same phenotype as that of uncultured, directly extracted disc cells), cells were treated for 48 hours with either 10 ng/ml IL-1α or 10 ng/ml IL-1β, or were left untreated to serve as controls; all treatments were performed in triplicate. Following treatment, RNA was extracted using Trizol reagent (Gibco). Prior to Trizol extraction, alginate beads were washed in 0.15 M NaCl and dissolved in dissolving buffer (55 mM sodium citrate, 30 mM EDTA, 0.15 M NaCl; pH 6) at 37°C for 15 min and then were subsequently digested in 0.06% w/v collagenase type I (Gibco) for 30 min to allow digestion of matrix. Following RNA extraction, reverse transcription was performed as described previously.

#### Real-time PCR

Real-time PCR was used to investigate the effects of IL-1 on a range of targets, namely, the members of the IL-1 family (IL-1α, IL-1β, IL-1Ra, and IL-1RI), matrix-degrading enzymes (MMP-3, MMP-13, ADAMTS-4, and ADAMTS-5), matrix proteins (aggrecan and collagen types I and II), and two SOX genes (6 and 9). Primers and Probes for all of these targets were designed using the Primer Express computer program (Applied Biosystems, Warrington, UK), using the rules of primer design recommended by Applied Biosystems. The total gene specificity of the nucleotide sequences chosen for the primers and probes were confirmed by BLAST searches (GenBank database sequences). The nucleotide sequences of the oligonucleotide hybridization primers and probes are shown in Table 2. Primers and probes were purchased from Applied Biosystems, as were pre-designed primers and probe (PDAR) for human glyceraldehyde-3-phosphate dehydrogenase (GAPDH). For each set of primers and probes, the efficiency of the amplification was assessed using template titrations as recommended by Applied Biosystems.

PCR reactions were then performed and monitored using the ABI Prism 7700 Sequence Detection System (Applied Biosystems). The PCR master mix was based on the AmpliTaq Gold DNA polymerase (Applied Biosystems). cDNA samples (2.5 μl in a total of 25 μl per well) were analysed in duplicate; primers were used at a concentration of 900 nmol/l and probe at 250 nmol/l. After real-time amplification, the ABI 7700 expressed the data as an amplification plot, from which a baseline was set from cycle number 3 upto a few cycles before the first visible amplification. In addition to the baseline, the threshold was set at a level above background levels and within the exponential phase of the PCR amplification. The same threshold was used for a target between runs. The Ct values for each target gene (cycle at which the set threshold is reached) were then exported into an Excel file, where analysis was performed using the 2^-ΔΔCt ^method, using GAPDH as the housekeeping gene, and normalized to untreated controls [39].

#### Statistical analysis

One-way ANOVA and Tukey post hoc tests were used to compare cells treated with IL-1 with those untreated samples. To perform this analysis, 2^-ΔΔCt ^for each sample was calculated using an average of untreated control ΔCt values to generate the relative gene expression for each sample, including all control values. These values were then used in ANOVA and post hoc tests; each treatment group was compared with untreated controls.

## Results

### Immunohistochemical localisation

**Figure 1:**
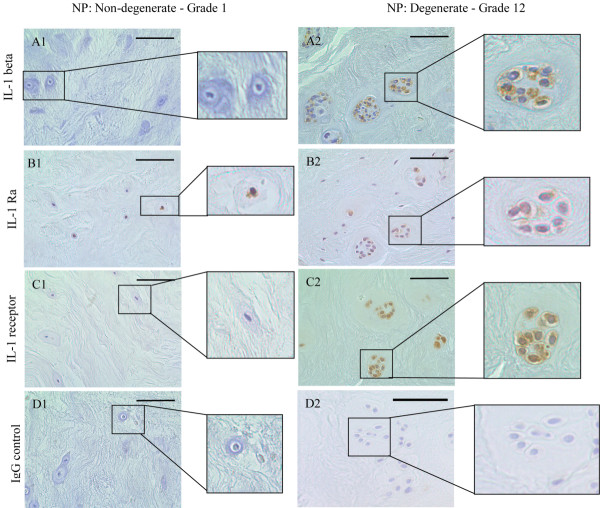
Examples of imunohistochemical staining for the IL-1 family. IL-1β (row A), IL-1Ra (row B), and IL-1 receptor, type I (row C) in grade-1 non-degenerate discs (column 1) and grade-12 degenerate discs (column 2), IgG controls (row D) were all negative. Immunopositivity is revealed by brown staining. N.B In non-degenerate discs, no cell clusters were seen and little immunopositivity was observed in the single cells. In degenerate discs, a large number of cell clusters were observed, which were predominately immunopositive. Bars = 570 μm.

Immunoreactivity for the five molecules studied (IL-1α, IL-1β, IL-1Ra, IL-1RI, and ICE) was seen in degenerate and non-degenerate IVDs. The immunostaining was generally restricted to the cytoplasm of native disc cells in normal and degenerate discs (Fig. 1). Staining was particularly prominent in the cytoplasm of the chondrocyte-like cells of the NP and IAF. No significant difference was observed between the proportions of cells in the NP and IAF reacting for IL-1α, IL-1Ra, and ICE (*P *= 1.525, 0.870, and 0.639, respectively). IL-1β and IL-1RI immunopositive cells were more frequent in the NP than the IAF (IL-1β, *P *< 0.05; IL-1RI, *P *< 0.05)).

**Figure 2:**
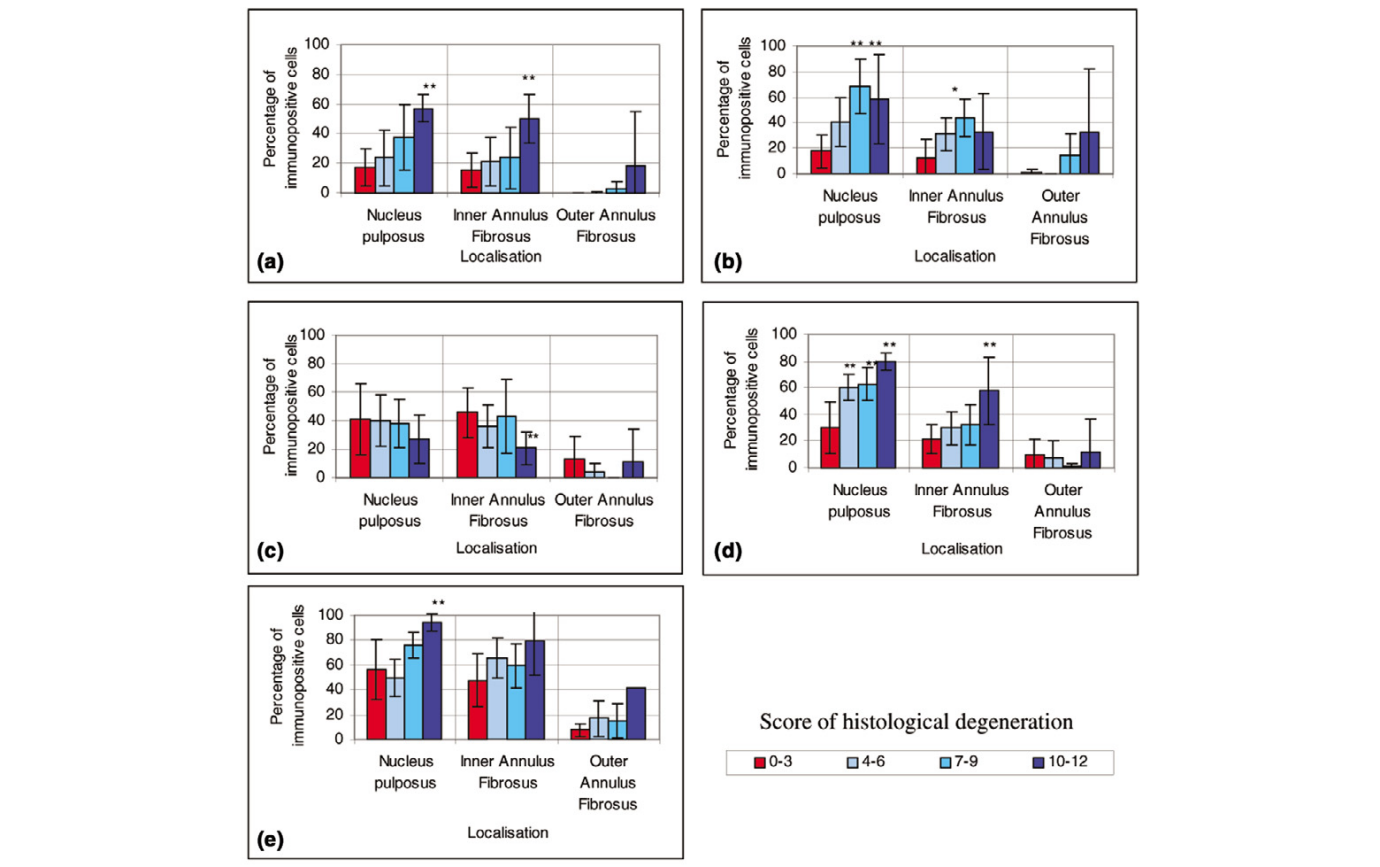
Immunopositive staining for the IL-1 family in human intervertebral discs. Numbers of cells with immunopositivity for IL-1α **(a)**, IL-1β **(b)**, IL-1 receptor antagonist **(c)**, IL-1 receptor, type I **(d)**, and IL-1β-converting enzyme **(e)**, according to place of origin in the disc and grade of intervertebral disc degeneration (*n *= 30). Data are presented as means ± 2 standard errors (as a representative of 95%CI). *P < 0.1,; **P < 0.05

IgG controls were always negative and all positive controls showed strong immunoreactivity (Fig. 1). No immunopositivity was observed in the matrix of the IVDs or in blood vessels, with the exception of immunopositivity for ICE, which showed some staining in the matrix and blood vessels of the most degenerate discs (histological degenerative scores 10 to 12). Although cells in the OAF did show reactivity for all molecules, the proportion was always significantly lower than in the NP and IAF (All targets *P *< 0.05) (Fig. 2).

#### Immunohistochemical staining and quantification of immunopositive cells

The most prominent aspects of the immunophenotype of non-degenerate discs (histological degeneration scores 0 to 3) included: little immunoreactivity for any of the five molecules in the OAF; low proportions of cells immunopositive for IL-1α, IL-1β, and IL-1RI in the NP and IAF (approximately 20%); the presence of IL-1Ra immunopositive cells in every disc, with high proportions of cells of up to 40% showing immunopositivity in the NP and IAF; and high numbers of cells in the NP and IAF also showing immunopositivity for ICE (60%) (Fig. 2).

In the degenerate IVDs (histological degenerative scores 4 to 12), the immunophenotype of cells differed in two ways from cells in non-degenerate discs (scores 0 to 3). Firstly, the proportion of cells immunopositive for IL-1α, IL-1β, IL-1RI, and ICE in both the NP and IAF was two or three times that in cells from non-degenerate IVDs, and this immunopositivity increased with the severity of degeneration. The difference between the degenerate and non-degenerate samples was significant in the NP and IAF in a number of stages of histological degeneration: IL-1α (NP and IAF: non-degenerate vs degenerate grades 10–12, *P *< 0.05); IL-1β (NP and IAF: non-degenerate vs three degrees of degeneration (scores 4 to 6, 7 to 9, and 10 to 12), all *P *< 0.05); IL-1RI (NP: non-degenerate vs three degrees of degeneration, all *P *< 0.05; IAF: non-degenerate vs severe grades of degeneration (scores 10 to 12), *P *< 0.05); ICE (NP: non-degenerate vs severe grades of degeneration, *P *< 0.05; IAF: non-degenerate vs severe grades of degeneration, *P *< 0.05). Secondly, similar numbers of IL-1Ra-immunopositive disc cells were seen in levels of degeneration scoring 4 to 6 and 7 to 9 and in non-degenerate discs, but in severe degeneration (scores 10 to 12), a significant decrease in the proportion of cells with IL-1Ra-immunopositivity was seen compared toI that seen in non-degenerate discs (*P *< 0.05) (Fig. 2).

### Assessment of redifferentiated state in alginate

**Figure 3:**
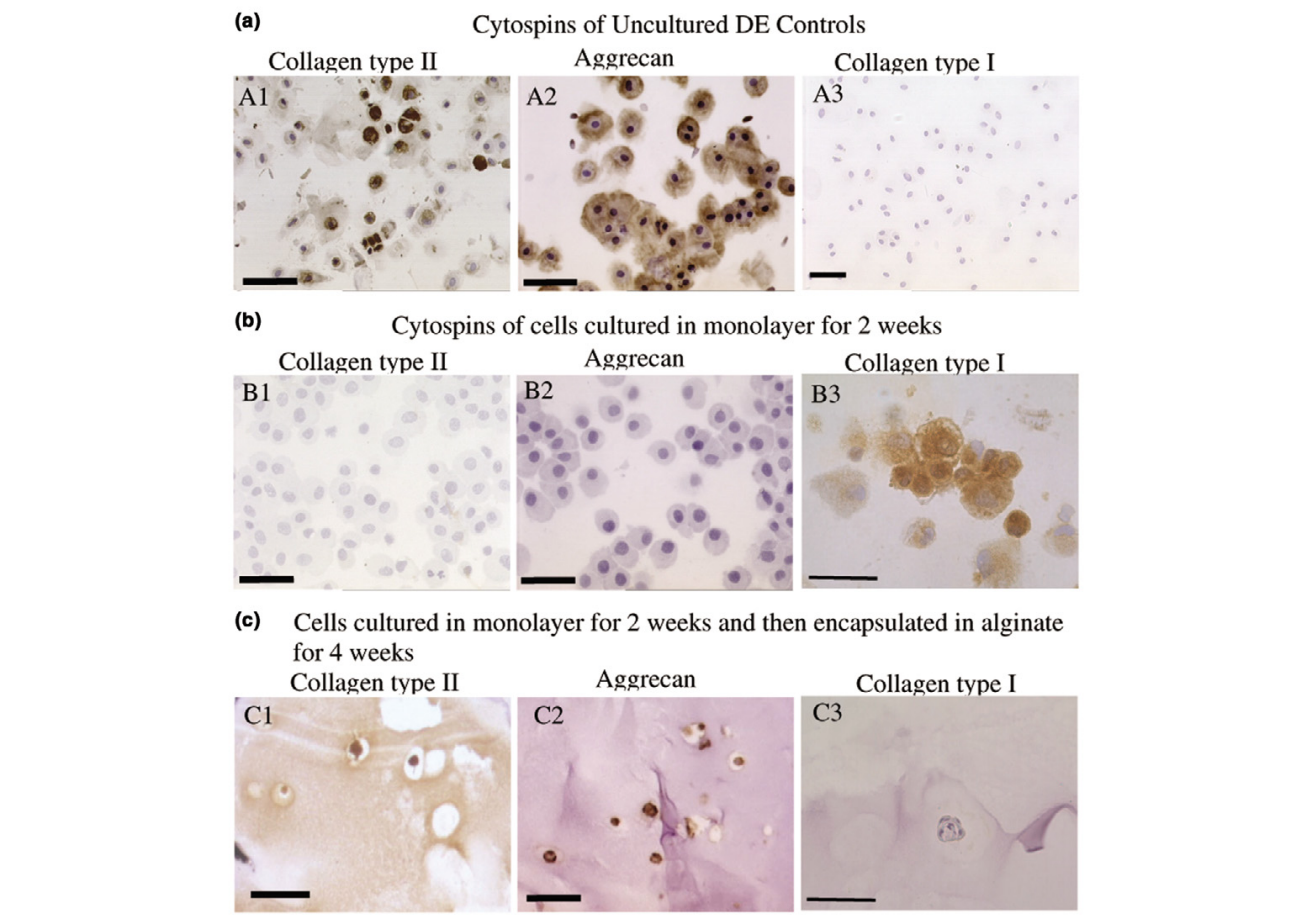
Immunopositive staining for phenotypic markers in chondrocyte-like cells from human intervertebral discs. Immunohistochemical staining for collagen type II, aggrecan, and collagen type I in uncultured directly extracted cells **(a)**, cells cultured in monolayer for 2 weeks and cytospun prior to staining **(b)**, and cells cultured in monolayer for 2 weeks prior to transfer to alginate and then cultured for a further 4 weeks **(c)**. Immunopositivityis revealed bybrown staining. Data shown are from cells derived from degenerate discs, but results were similar in non-degenerate discs. Bars = 570 μm. DE, directly extracted.

NP and AF cells directly extracted from IVD tissue showed similar morphology and phenotypic characteristics. Morphologically, the cells were small and rounded, often (in cells from degenerate discs) localized in clusters. Immunopositivity for aggrecan and collagen type II was seen, but no cells immunopositive for collagen type I were observed (Fig. 3a). In monolayer, these cells adhered and spread, developing a fibroblastic morphology, together with loss of immunopositivity for aggrecan and collagen type II, and they expressed collagen type I protein (Fig. 3b). However, when transferred to alginate and cultured for 4 weeks, these cells regained their rounded morphology and began to produce aggrecan and collagen type II protein, and lost their immunopositivity to collagen type I (Fig. 3c), resembling the immunohistochemical profile of uncultured, directly extracted cells. Gene expression analysis showed a similar pattern to protein production in monolayer and alginate cultures, with 4 weeks' culture in alginate required before gene expression levels returned to that seen in uncultured, directly extracted cells (*P *> 0.05) (data not shown). No significant difference was observed in the re-differentiation potential of cells extracted from NP or from AF cells, or between cells extracted from non-degenerate or from degenerate IVDs.

#### Effect of IL-1 on human IVD cells

Interleukin 1 treatment (IL-1α and IL-1β) of the four cell types/origins (degenerate and non-degenerate cells, from AF or NP) resulted in altered in expression of genes for matrix molecules and matrix-degrading enzymes. The responses of cells to IL-1α and IL-1β were similar, and hence only the effects of IL-1β are detailed here. Although it can be generally summarized that IL-1 caused an increase in gene expression for matrix-degrading enzymes, particularly in cells derived from the degenerate NP, and caused a decrease in normal matrix molecule gene expression in cells derived from normal discs, the pattern was complex and dependent upon the origin of the cells (Table 3).

#### Effect of IL-1 on degradative enzymes

**Figure 4:**
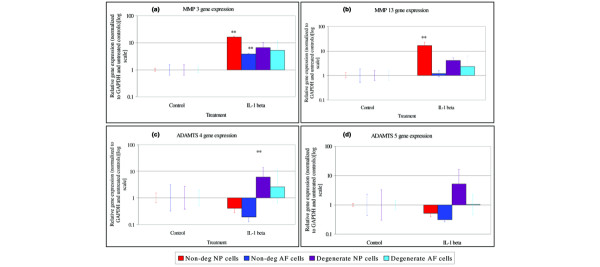
Effect of IL-1 on MMP and ADAMTS gene expression in cells from human intervertebral discs. Relative gene expression was normalized to that of the GAPDH (glyceraldehyde-3-phosphate dehydrogenase) housekeeping gene and untreated controls (hence control is graphed at 1 on the log scale) for matrix metalloproteinase (MMP)-3 **(a)**, MMP-13 **(b)**, ADAMTS (a disintegrin and metalloproteinase with thrombospondin motifs)-4 **(c)**, and ADAMTS-5 **(d) **following IL-1β treatment of cells from two regions of non-degenerate (non-deg) (*n *= 6) and degenerate (*n *= 24) discs. **P < 0.05. AF, annulus fibrosus; NP, nucleus pulposus.

Following treatment with IL-1, an increase in MMP-3 gene expression was seen in the four cell types investigated (though the increase was significant only in cells derived from the non-degenerate NP and AF (*P *< 0.05)) (Fig. 4a). An increase in MMP-13 gene expression was also observed, but only in cells derived from the NP, with significance achieved in cells from non-degenerate discs (*P *< 0.05) (Fig. 4b). Aggrecanase (ADAMTS-4 and -5) gene expression was increased in cells derived from the NP of degenerate discs. This was significant only for ADAMTS-4 (*P *< 0.05). In cells derived from the non-degenerate discs, a slight, nonsignificant decrease in aggrecanase gene expression was observed (Fig. 4c,d).

#### Effect of IL-1 on matrix molecules

**Figure 5:**
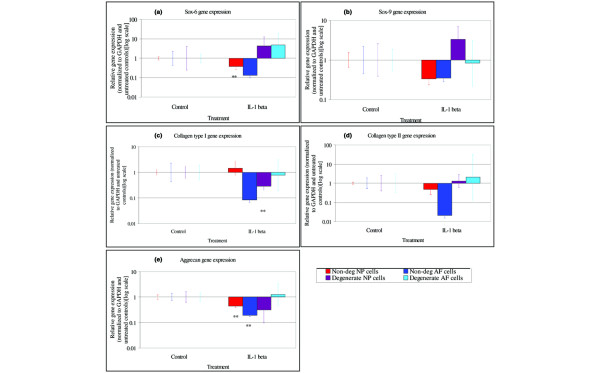
Effect of IL-1 treatment on matrix gene expression in cells from human intervertebral discs. Relative gene expression was normalized to the glyceraldehyde-3-phosphate dehydrogenase (GAPDH) housekeeping gene and untreated controls (hence control is graphed at 1 on the log scale) for SOX6 **(a)**, SOX9 **(b)**, collagen type I **(c)**, collagen type II **(d)**, and aggrecan **(e) **following IL-1β treatment of disc cells from two regions of non-degenerate (non-deg) (*n *= 6) and degenerate (*n *= 24) discs. **P < 0.05. AF, annulus fibrosus; NP, nucleus pulposus.

IL-1 treatment of cells derived from non-degenerate discs resulted in a decrease in both SOX6 and SOX9 gene expression. However, this achieved significance only for SOX6 (*P *< 0.05). No real effect was observed on SOX6 and SOX9 gene expression in cells derived from degenerate discs (Fig. 5a,b). A decrease was also observed in expression of the gene for collagen type I in cells derived from non-degenerate AF and degenerate NP; however this was significant only in cells derived from degenerate NP (*P *< 0.05) (Fig. 5c). The expression of the genes for collagen type II and aggrecan were decreased by IL-1 treatment of cells derived from the non-degenerate disc, although this decrease was only significant for aggrecan (Fig. 5d,e).

#### IL-1 regulation

**Figure 6:**
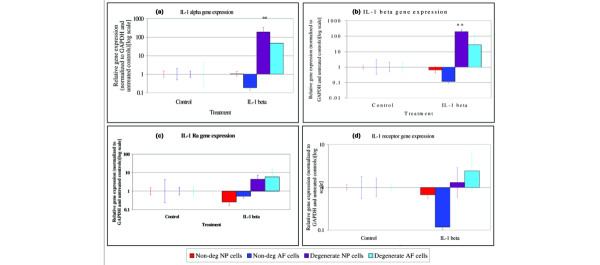
Effect of IL-1 treatment on the IL-1 family gene expression in human intervertebral disc cells. Relative gene expression was normalized to glyceraldehyde-3-phosphate dehydrogenase (GAPDH) housekeeping gene and untreated controls (hence control is graphed at 1 on the log scale) for IL-1α **(a)**, IL-1β **(b)**, IL-1 receptor antagonist (IL-1Ra) **(c)**, and IL-1 receptor, type I (IL-RI) **(d) **following IL-1β treatment of disc cells from two regions of non-degenerate (non-deg) (*n *= 6) and degenerate (*n *= 24) discs. **P < 0.05.

IL-1 treatment of cells derived from the degenerate but not the non-degenerate disc resulted in a 100-fold increase in IL-1α and IL-1β gene expression, which reached significance in cells derived from the NP (*P *< 0.05) (Fig. 6a,b). No real trend was observed in IL-1Ra gene expression after treatment with IL-1 (Fig. 6c). A 10-fold decrease in IL-1 receptor gene expression was observed in cells derived from the non-degenerate AF, but this was not significant and no effect was observed on the other cell types (Fig. 6d).

## Discussion

In this study, we investigated whether in IVD degeneration there is local production of the cytokine IL-1 and whether IL-1 could induce the cellular changes characteristic of IVD degeneration. To date, the production of IL-1 by human IVD cells has been shown only in cells derived from herniated tissue [18,19,29,30,40]. However, herniated tissue is not representative of native disc tissue and is usually contaminated with inflammatory cells. For example, Doita and colleagues localized production of IL-1 to infiltrating mononuclear cells within sequestered and extruded disc tissue but did not show any significant immunodetectable IL-1 in connective tissue cells in the displaced IVDs [29]. The current study is the first to investigate protein production and localization of IL-1 in intact, non-degenerate and degenerate human IVDs themselves, as opposed to herniated disc tissue.

This study has shown that both isoforms of IL-1 (IL-1α and IL-1β) are produced by the chondrocyte-like cells of the NP and IAF (but not blood vessels or fibroblast like cells in the OAF) of non-degenerate and degenerate IVDs. Furthermore, chondrocyte-like cells in non-degenerate IVDs express and produce the active receptor IL-1RI, indicating that they can respond to IL-1. Importantly, in degenerate IVDs there is a significant increase in IL-1RI-immunopositive chondrocyte-like cells by comparison with non-degenerate IVDs, indicating an increased responsiveness to IL-1; and there are increased numbers of chondrocyte-like cells expressing ICE, an enzyme required to convert the inactive pro-IL-1β into its active form [41].

This study demonstrated IL-1Ra protein localization to cells in both non-degenerate and degenerate human IVDs. The production of IL-1Ra in the non-degenerate disc demonstrates a means of regulating IL-1. Within most clinical conditions involving IL-1, an increase in IL-1Ra production is considered an excellent marker of disease, and often a better indicator than IL-1 itself [42]. For example, in rheumatoid arthritis, raised IL-1Ra production is considered to be a natural compensatory mechanism to counter the activity of IL-1 [43]. In the current study, a marked increase in the proportion of cells immunoreactive for IL-1 were found in degenerate than in non-degenerate IVDs, but no similar increase in IL-1Ra-immunopositive cells was observed, indicating an imbalance in the local production of IL-1 and IL-1Ra and failure of the normal compensatory mechanism associated with increasing local production of IL-1. When coupled with an increase in IL-1 receptor and ICE with increasing degeneration, the net effect would be the initiation and perpetuation of an IL-1-mediated response.

Having established a basis for a functional excess of IL-1 in degenerate IVDs, we then investigated the role of IL-1 in the processes that characterize disc degeneration, namely, decreased matrix synthesis and increased production of MMPs and ADAMTS-4 [3-6]. This is the first time such a comprehensive study has been undertaken in human IVD cells. Such limited studies as have been conducted previously on IVD cells have focused on cell monolayers and have not used human cells [24,26,27]. However, it is well known that cells in monolayer culture dedifferentiate and therefore effects may be very different from those *in vivo*. Culture of cells in 3D gels such as alginate allows the phenotype of IVD chondrocyte-like cells to be maintained [37,44-46]. To date, only two studies have investigated the effects of IL-1 in such systems, one using ovine IVD cells [25] and the other, rabbit IVD cells [28]. This is the first reported study to investigate the effects of IL-1 on human disc cells cultured in 3D gels.

### Effect of IL-1 on degradative enzymes

In the current study, MMP-3 mRNA expression was increased in NP and AF cells derived from non-degenerate and degenerate IVDs after IL-1 treatment, a phenomenon reported in rabbit disc cells cultured in monolayer [27] and ovine NP cells cultured in agarose [25]. Therefore, *in vitro *IL-1 causes an increase expression of MMP-3, an enzyme increased in the degenerate disc [6]

Treatment of NP (but not AF) cells from degenerate and non-degenerate IVDs with IL-1 resulted in significant increases in gene expression of MMP-13 (an MMP with high affinity for type II collagen), a finding not previously reported in disc cells, although it has been shown in articular chondrocytes [16,47,48]. We have previously shown that immunodetectable MMP-13 protein is present in significant amounts in IVDs, with the highest immunopositivity in the NP of degenerate discs [6], an area of the IVD containing the highest concentration of collagen type II.

ADAMTS-5 gene expression was not significantly altered by IL-1 treatment. However, such treatment did result in an increase in the gene expression of the aggrecanase ADAMTS-4 in cells derived from degenerate NP. *In vivo*, the NP contains the highest concentration of aggrecan in the IVD. The response of cells derived from degenerate NP to IL-1 to up-regulate ADAMTS-4 indicates that *in vivo *a local increase in the concentration of IL-1 might lead to the dehydration and loss of height characteristic of IVD degeneration, through the production of aggrecanases by local cells. We have previously shown an increase in ADAMTS-4 production by the cells of degenerate discs, especially in the NP [6], which, interestingly, were the same discs shown in this study to produce high levels of IL-1 agonists.

### Effect of IL-1 on matrix molecules

Degeneration of the IVD is associated with altered collagen production by IVD cells, with a switch in synthesis from type II to type I collagen in the IAF and NP [7]. Proteoglycan production is also altered, with decreased aggrecan [8] and increased production of versican, biglycan, and decorin [9,10]. IL-1 has been implicated in changes in matrix synthesis during degradation of articular cartilage, with studies showing a down-regulation of the genes for SOX9 and collagen type IIa1 [49], aggrecan, collagen types II and XI, and link proteins [48,50], and inhibition of normal matrix assembly [51]. The few studies performed on IVD cells have shown that IL-1 treatment also causes a decrease in proteoglycan and collagen II production in animal cells [24,26-28]. The current study demonstrates that IL-1 decreases expression of the gene for SOX6 by cells of the non-degenerate IVD. SOX6 (usually in combination with SOX9, which was also decreased by IL-1 [albeit not significantly] in this study) determines the chondrogenic phenotype [52]. Such results suggest that IL-1 can inhibit the innate regulator of the chondrocyte-like cells' chondrogenic phenotype, resulting in IVD cells, particularly in the NP, that develop a less differentiated and more fibroblastic phenotype. This inhibition of the SOX genes might lead to the altered collagen and aggrecan synthesis typical of IVD degeneration [7-10,53]. The current study also demonstrated that IL-1 inhibited expression of the genes for collagen types I and II and for aggrecan. This would mean that within the NP, at least, IL-1 can exert its effects on biosynthesis in the same way as it does in articular cartilage [49].

Interestingly, this study has also shown that the cells derived from degenerate and non-degenerate discs respond differently to IL-1. In particular, cells from degenerate IVDs respond to IL-1 with a further increase in IL-1 gene expression (i.e. there is a positive autocrine effect), while cells from non-degenerate discs showed a decrease, suggesting that the normal homeostatic response to IL-1 is replaced in the degenerate IVD by a positive feedback loop. Such a phenomenon has also been reported in human skin fibroblasts treated with IL-1 [54]. This positive feedback loop in degenerate disc cells clearly distinguishes them from non-degenerate disc cells.

## Conclusion

We have shown that IL-1 is produced in the degenerate IVD. It is normally synthesized by the native chondrocyte-like cells, but in the non-degenerate IVD there is a balance between IL-1 and its inhibitor, IL-1Ra, ensuring that matrix homeostasis is maintained. Treatment of human IVD cells with IL-1 disturbs the normal balance of catabolic and anabolic events, with the result that degrading enzymes are increased and the expression of genes for matrix proteins are decreased, responses that correspond to the alterations of cell biology that characterize IVD degeneration. In addition, the immunohistochemical data from this study demonstrated that although numbers of cells with immunopositivity for the IL-1 agonists increased with degeneration, no such increase was seen in the numbers of cells with immunopositivity for IL-1Ra. This finding suggests that the normal inhibitory mechanism fails in disc degeneration, with a loss in the balance of IL-1 agonists to antagonists, allowing IL-1 to elicit and perpetuate a response. We have also shown that cells from non-degenerate and degenerate discs respond differently to IL-1. In particular, IL-1 causes cells from degenerate IVDs to synthesize more IL-1, with the potential to induce accelerating degeneration.

This study has shown how IL-1, a naturally occurring cytokine within the IVD, could, through an imbalance between it and its inhibitor, play a role in the pathogenesis of IVD degeneration and therefore be an important therapeutic target for preventing and reversing disc degeneration.

## Abbreviations

ADAMTS = a disintegrin and metalloproteinase with thrombospondin motifs; AF = annulus fibrosus; DMEM + F12 = Dulbecco's modified Eagle's medium and Ham's F12 nutrient medium; EDTA = ethylenediaminetetraacetic acid; GAPDH = glyceraldehyde-3-phosphate dehydrogenase; H&E = haematoxylin and eosin; IAF = inner annulus fibrosus; ICE = IL-1β-converting enzyme; IL-1 = interleukin-1; IL-1Ra = IL-1 receptor antagonist; IL-RI = IL-1 receptor, type I; IVD = intervertebral disc; MMP = matrix metalloproteinase; NP = nucleus pulposus; OAF = outer annulus fibrosus.

## Competing interests

The author(s) declare that they have no competing interests.

## Authors' contributions

CLM participated in the design of the study, performed all laboratory work and analysis, and drafted the manuscript. AJF conceived the study and participated in its design and coordination. JAH conceived the study, participated in its design and coordination, and assisted in its analysis. All authors read and approved the final manuscript.

## Figures and Tables

**Table 1:** Patient details and grades of disc degeneration in tissues used for immunohistochemical analysis

Laboratory number	Source of tissue	Sex	Age (y)	Clinical diagnosis	Disc level	Histological grade
1	Post-mortem	Male	53	No data	L4/5	1
2	Post-mortem	Male	53	No data	L5/S1	1
3	Surgical	Male	44	Relatively normal	L4/5	1
4	Surgical	Male	47	Relatively normal	L4/5	2
5	Post-mortem	Male	75	No data	L5/S1	3
6	Surgical	Male	?	Disc degeneration	L5/S1	3
7	Surgical	Male	48	Disc degeneration	L4/5	3
8	Surgical	Male	64	Disc degeneration	L5/S1	3
9	Surgical	Male	46	Disc degeneration	L5/S1	4
10	Surgical	Male	21	Disc degeneration	L5/S1	4
11	Surgical	Female	36	Disc degeneration	L5/S1	4
12	Surgical	Male	39	Disc degeneration	L4/5	5
13	Surgical	Female	32	Disc degeneration	L5/S1	5
14	Surgical	Female	36	Disc degeneration	L4/5	5
15	Surgical	Male	25	Disc degeneration	L4/5	5
16	Surgical	Female	35	Disc degeneration	L4/5	6
17	Surgical	Male	40	Disc degeneration	L4/5	6
18	Post-mortem	Female	73	No data	L5/S1	6
19	Surgical	Male	25	Disc degeneration	L5/S1	6
20	Surgical	Female	55	Disc degeneration	L5/S1	7
21	Post-mortem	Female	?	No data	L4/5	7
22	Surgical	Female	58	Disc degeneration	L4/5	7
23	Surgical	Male	34	Disc degeneration	L4/5	8
24	Surgical	Female	24	Disc degeneration	L5/S1	8
25	Surgical	Female	33	Disc degeneration	L5/S1	9
26	Post-mortem	Female	73	No data	L4/5	9
27	Surgical	Male	68	Disc degeneration	L5/S1	10
28	Post-mortem	?	47	No data	L5/S1	10
29	Post-mortem	?	47	No data	L5/S1	11
30	Surgical	Male	39	Disc degeneration	L4/5	12

?, not known.

**Table 2:** Real-time PCR probes and details of primers

Target	Forward primer	Probe	Reverse primer	Threshold
GAPDH	PDAR	PDAR	PDAR	0.047
Collagen type I	5' CAG CCG CTT CAC CTA CAG C 3'	5' CCG GTG TGA CTC GTG CAG CCA TC 3'	5' TTT TGT ATT CAA TCA CTG TCT TGC C 3'	0.078
Collagen type II	5' GGC AAT AGC AGG TTC ACG TAC A 3'	5' CCG GTA TGT TTC GTG CAG CCA TCC T 3'	5' CGA TAA CAG TCT TGC CCC ACT T 3'	0.100
Aggrecan	5' TCG AGG ACA GCG AGG CC 3'	5' ATG GAA CAC GAT GCC TTT CAC CAC GA 3'	5' TCG AGG GTG TAG CGT GTA GAG A 3'	0.050
SOX9	5' GAC TTC CGC GAC GTG GAC 3'	5' CGA CGT CAT CTC CAA CAT CGA GAC 3'	5' GTT GGG CGG CAG GTA CTG 3'	0.0562
SOX6	5' CCG TGA GAT AAT GAC CAG TGT TAC TT 3'	5' AAC CCC AGA GCG CCG CAA A 3'	5' GTC CAC CAC ATC GGC AAG AC 3'	0.052
IL-1α	PDAR	PDAR	PDAR	0.107
IL-1β	PDAR	PDAR	PDAR	0.122
IL-1Ra	5' CCT GCA GGG CCA AGC A 3'	5' AGC CTC GCT CTT GGC AGG TAC TCA GT 3'	5' GCA CCC AAC ATA TAC AGC ATT CA 3'	0.122
IL-1RI	5' ATT TCT GGC TTC TAG TCT GGT GTT C 3'	5' ACT TGA TTT CAG GTC AAT AAC GGT CCC C 3'	5' AAC GTG CCA GTG TGG AGT GA 3'	0.163
MMP-3	5' TGA AGA GTC TTC CAA TCC TAC TGT TG 3'	5' CGT GGC AGT TTG CTC AGC CTA TCC AT 3'	5' CTA GAT ATT TCT GAA CAA GGT TCA TGC A 3'	0.108
MMP-9	5' CCC GGA GTG AGT TGA ACC A 3'	5' CCA AGT GGG CTA CGT GAC CTA TGA CAT CC 3'	5' CAG GAC GGG AGC CCT AGT C 3'	0.041
MMP-13	5' GGA CAA GTA GTT CCA AAG GCT ACA A 3'	5' CTC CAA GGA CCC TGG AGC ACT CAT GTT 3'	5' CTT TTG CCG GTG TAG GTG TAG ATA G 3'	0.108
ADAMTS-4	5' ACT GGT GGT GGC AGA TGA CA 3'	5' ATG GCC GCA TTC CAC GGT G 3'	5' TCA CTG TTA GCA GGT AGC GCT TT 3'	0.052
ADAMTS-5	5' GGA CCT ACC ACG AAA GCA GAT C 3'	5' CCC AGG ACA GAC CTA CGA TGC CAC C 3'	5' GCC GGG ACA CAC GGA GTA 3'	0.122

ADAMTS, a disintegrin and metalloproteinase with thrombospondin motifs; GAPDH, glyceraldehyde-3-phosphate dehydrogenase; IL-1Ra, IL-1 receptor antagonist; IL1-RI, receptor, type I; MMP, matrix metalloproteinase; PDAR pre-designed assay reagent.

**Table 3:** Effects of IL-1β on gene expression in cells from non- degenerate or degenerate intervertebral discs

Target	General trend	Tissues affected		Significant changes^a ^(*P *< 0.05)
			
		Origin of cells	Disease state	
MMP-3	Increase (5- to 10-fold)	NP, AF	N, D	Non-degenerate NP and AF
MMP-13	Increase (5- to 10-fold)	NP	N, D	Non-degenerate NP
ADAMTS-4	Increase (8-fold)	NP	D	Degenerate NP
ADAMTS-5	No real trend	-	-	None
SOX6	Decrease (3- to 9-fold)	NP, AF	N	Non-degenerate NP
SOX9	Decrease (3-fold)	NP, AF	N	None
Collagen I	Decrease (5- to 10-fold)	NP, AF	N, D	Degenerate NP
Collagen II	Decrease (5- to 50-fold)	AF	N, D	None
Aggrecan	Decrease (3- to 7-fold)	NP, AF	N, D	Non-degenerate NP and AF
IL-1α	Increase (100-fold)	NP, AF	D	Degenerate NP
IL-1β	Increase (100-fold)	NP, AF	D	Degenerate NP
IL-1Ra	No real trend	-	-	None
IL-1RI	Decrease (2- to 10-fold)	NP, AF	N	None

^a^Site of any significant change in gene expression. -, no effect seen; ADAMTS, a disintegrin and metalloproteinase with thrombospondin motifs; AF, annulus fibrosus; D, degenerate intervertebral disc; IL-1Ra, IL-1 receptor antagonist; IL1-RI, receptor, type I; MMP, matrix metalloproteinase; N, non-degenerate intervertebral disc; NP, nucleus pulposus.
